# Interfacial Resistive Switching of Niobium–Titanium Anodic Memristors with Self-Rectifying Capabilities

**DOI:** 10.3390/nano14040381

**Published:** 2024-02-19

**Authors:** Dominik Knapic, Alexey Minenkov, Elena Atanasova, Ivana Zrinski, Achim Walter Hassel, Andrei Ionut Mardare

**Affiliations:** 1 Institute of Chemical Technology of Inorganic Materials, Johannes Kepler University Linz, Altenberger Str. 69, 4040 Linz, Austria; dominik.knapic@jku.at (D.K.); elena.atanasova@jku.at (E.A.); zrinski.ivana@gmail.com (I.Z.); achimwalter.hassel@jku.at (A.W.H.); 2 Christian Doppler Laboratory for Nanoscale Phase Transformations, Center for Surface and Nanoanalytics, Johannes Kepler University Linz, Altenberger Str. 69, 4040 Linz, Austria; oleksii.minienkov@jku.at; 3 Faculty of Medicine and Dentistry, Danube Private University, Steiner Landstraße 124, 3500 Krems an der Donau, Austria

**Keywords:** anodic memristor, Nb-Ti compositional library, interfacial, neuromorphic computing, self–rectifying

## Abstract

A broad compositional range of Nb-Ti anodic memristors with volatile and self-rectifying behaviour was studied using a combinatorial screening approach. A Nb-Ti thin-film combinatorial library was co-deposited by sputtering, serving as the bottom electrode for the memristive devices. The library, with a compositional spread ranging between 22 and 64 at.% Ti was anodically oxidised, the mixed oxide being the active layer in MIM-type structures completed by Pt discreet top electrode patterning. By studying *I–U* sweeps, memristors with self-rectifying and volatile behaviour were identified. Moreover, all the analysed memristors demonstrated multilevel properties. The best-performing memristors showed HRS/LRS (high resistive state/low resistive state) ratios between 4 and 6 × 10^5^ and very good retention up to 10^6^ successive readings. The anodic memristors grown along the compositional spread showed very good endurance up to 10^6^ switching cycles, excluding those grown from alloys containing between 31 and 39 at.% Ti, which withstood only 10 switching cycles. Taking into consideration all the parameters studied, the Nb-46 at.% Ti composition was screened as the parent metal alloy composition, leading to the best-performing anodic memristor in this alloy system. The results obtained suggest that memristive behaviour is based on an interfacial non-filamentary type of resistive switching, which is consistent with the performed cross-sectional TEM structural and chemical characterisation.

## 1. Introduction

The field of complementary metal-oxide semiconductor (CMOS) technology and related architectures, including memristors, has seen a surge in research activity in recent years. A memristor [1] is a basic two-terminal passive electronic component that was first theorised by Chua in 1971 and was developed experimentally by Hewlett Packard Labs more than three decades later [2]. Depending on their behaviour upon the suppression of an externally applied electrical field, memristors can be defined as non-volatile when they maintain (remember) their set resistance state and volatile when they return to their initial state [3]. Additionally, volatile memristors can be classified by their switching behaviour as digital (threshold) when they show an abrupt switching complying with an on/off process and analogue if they show a gradual transition through multiple resistive states [2]. The switching mechanism of digital memristors is usually filamentary, while analogue ones may have either filamentary, with parallel filaments defining the resistive levels [4,5], or nonfilamentary mechanisms, based on an interfacial switching mechanism [6]. With its unique volatile or non-volatile resistive switching behaviour, this circuit element (the fourth most common apart from the inductor, capacitor, and resistor) can be used in a variety of modern applications such as memory devices [7,8,9,10,11,12,13], sensing [14,15,16], image processing [17,18], and neuromorphic computing [19,20,21].

While non-volatile memristors mimic biological processes associated with learning and memorising, volatile memristors mimic forgetting [22]. The volatile switching behaviour is especially suitable for synaptic devices for neuromorphic computing, the imitation of nociceptors, and physical reservoirs for handling temporal data in reservoir computing [3,23,24]. The essential part of the brain architecture that is responsible for the mammalian ability to learn, memorise, and forget information is a synapse, a two-terminal structure which connects two neurons. Memristors are used as an electronic equivalent of a biological synapse in artificial neuromorphic networks [3,22]. Furthermore, volatile memristors are often used in a one-rectifier (diode), one-resistor (1D1R), or one-rectifier (diode)—one-memristor configuration (1D1M) to reduce sneak currents in crossbar array structures [23,25,26]. Self-rectifying memristors [27,28] have an integrated rectifier in their oxide due to the formation of a Schottky barrier. Such a structure can usually be obtained by the deposition of two different metal oxides [29] or by nanostructuring the active layer [27]. However, a rather new fabrication route involving the anodic oxidation of the metallic bottom electrode was shown to lead to various unexpected and promising intrinsic effects, facilitating defect engineering. Such an approach will be applied in this work. Unlike conventional memristors, where the oxide is deposited directly on the bottom electrodes, anodic memristors [13,30,31,32] are produced through the anodization of the bottom electrode. The oxide growth from a parent metal directly influences the metal/oxide interface and may play a role in the final device’s behaviour. The anodic formation of mixed oxides from metallic-bottom-electrode alloys provides a simplified, more direct route for obtaining the metal–insulator–metal (MIM) structures needed for self-rectifying memristors.

In the current study, a combinatorial approach was utilised to investigate anodically produced memristors based on Nb-Ti thin-film alloys. The impact of the Nb-Ti parent metal’s composition on the electrical behaviour of their mixed anodic oxides was investigated for screening possible memristive improvements as compared to pure Nb and Ti oxides. Nb was chosen due to its already reported potential for neuromorphic application [33,34,35] and Ti because it is already a well-studied and understood material for memristors [13,31,32]. Compositional spreads enable the study of a large number of alloys efficiently, making it possible to find the composition that is most suitable for a specific purpose [36], e.g., the memristor with the best properties within a given compositional range. Along the library, electrical screening was performed, characterising the memristive retention (reading) and endurance (writing). The best-performing anodic memristor was characterised on anatomical scale to conclude upon its switching mechanism and predominant conduction type.

## 2. Materials and Methods

### 2.1. Fabrication of Nb-Ti Anodic Memristors

**Figure 1:**
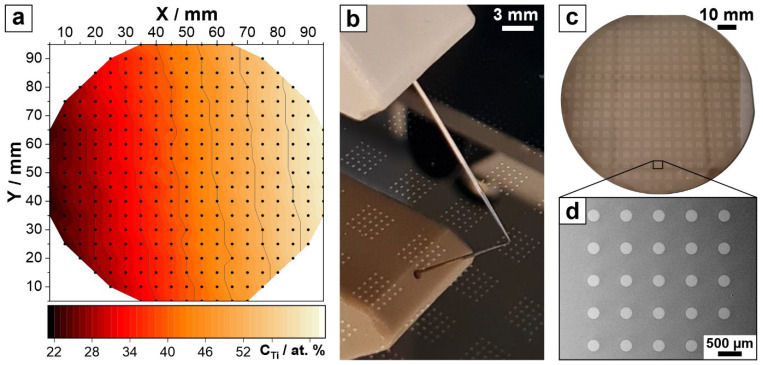
(**a**) Compositional gradient of the Nb-Ti library on a Si wafer. (**b**) Addressing one memristor from a cluster by contacting its top electrode. (**c**,**d**) Views of memristor clusters.

The co-deposition of Nb-Ti was performed by sputtering, producing a Nb-Ti combinatorial library. An ultra-high vacuum system (Mantis Deposition, United Kingdom), maintaining a base pressure around 10^−6^ Pa, was utilised. Thin films of Nb-Ti were sputtered in DC mode using high-purity Nb and Ti targets (99.95% Demaco, The Netherlands) in an argon atmosphere at 5 × 10^−1^ Pa onto Si substrates that had been preoxidized at 950 °C for 24 h. To ensure a wide compositional range, the targets were positioned opposite each other on Si wafers, with a distance of 13 cm from the substrate. Power levels ranging from 25 W to 80 W were applied to the Nb and Ti targets (both 50 mm in diameter) to control the total compositional range. The compositional gradient shown in Figure 1a was determined using a self-developed scanning energy-dispersive X-ray spectroscopy (SEDX) system within the same ultra-high vacuum chamber cluster. The system, equipped with a Si drift detector (SDD) operated at 20 keV, scanned the entire wafer surface automatically and mapped the alloy compositions using IDFix software. The spread ranged from 22 to 64 at.% Ti along the Nb-Ti library. Wafers with Nb-Ti thin films were transferred directly to the SEDX chamber after co-sputtering without breaking the vacuum. An oxide film was grown anodically on the surface of the Nb-Ti combinatorial library in an electrochemical setup using the deposited metallic film as the working electrode, Ag|AgCl|3 M KCl as the reference electrode, and a platinum mesh as the counter electrode. The anodization was performed potentiodynamically by running *I–U* sweeps in phosphate buffer solution (PB) with a potential range from 0 to 8 V (vs SHE) at a scan rate of 100 mV s^−1^. The selection of the maximum potential value was aimed at an oxide film approximately 20 nm thick, based on oxide formation factors for both Nb and Ti. The electrolyte was prepared using analytical-grade chemicals (NaH_2_PO_4_ and Na_2_HPO_4_) to create a 1 M phosphate buffer (PB) with a pH of 7.0, following a standard recipe [37]. The memristors were finalised by depositing top electrodes through a Ni shadow mask (Mecachimique, France) with a sputtering Pt target (99.95% Demaco, The Netherlands). Platinum was chosen for its attractive electrical properties, which define very good electrodes, and due to its very good adhesion to Ti. Circular Pt electrodes, 100 nm thick and 200 μm in diameter, were deposited at room temperature. Approximately 300 memristors, organised in 5 × 5 electrode clusters within each 3 × 3 mm^2^ surface area, were produced on each Si wafer. This setup, along with the measured concentration gradient and memristors produced on a wafer, is shown in Figure 1.

### 2.2. Electrical Characterisation

Electrical measurements were conducted utilizing a Keithley 2450 SourceMeter Unit (SMU) linked to a custom-built Gantry robot. This robot incorporates precise XYZ translation stages and has two microscope cameras (Bresser, Germany) to enable both top (normal) and side (45° angle) views of the Pt electrodes. A force sensor, integrated into the Z stage, holds a tungsten needle with a tip diameter of 10 µm for establishing electrical contact with the top electrode of a targeted memristor (depicted in Figure 1b), maintaining a constant force of 15 ± 1 mN. The bottom metallic film is contacted by another needle, forming a firm connection with the Nb-Ti thin film at the Si wafer’s edge. The entire setup is controlled by LabView^®^ software tailored for executing current–voltage (*I–U*) sweeps, endurance, and retention tests. All experiments were conducted under ambient conditions (22 °C, 55% RH), with voltage applied to the Nb-Ti bottom electrode, while the top Pt electrode was grounded. Using the SMU, *I–U* sweeps were recorded, with the compliance current managed within the mA range. Endurance tests were measured up to 10^6^ switching cycles, biasing them at voltages corresponding to their switching voltages, *U*_set_ or *U*_reset_, at a frequency of 260 Hz. Endurance tests were conducted up to 10^6^ switching cycles by biasing the memristors at their voltages corresponding to switching to LRS or HRS (*U*_set_ and *U*_reset_) with a frequency of 260 Hz. Retention tests were performed by sequentially measuring many times the resistance of a device in either HRS or LRS. The device resistance was always measured by applying a voltage of 0.001 V. The LRS was always read at the minimum resistance value found at the RESET, and the HRS state was read at the maximum resistance value found at 0.1 V. The values of *U*_set_ and *U*_reset_ ranged between ±3 and ±4 V, respectively.

### 2.3. Imaging and Analysis Techniques

The surface microstructure of the Nb-Ti thin-film alloys along the library was characterised with a field emission scanning electron microscope (FE-SEM, Zeiss Leo 1550 VP, Jena, Germany). Images were acquired at a 3 kV acceleration voltage using the in-lens detector. With these experimental conditions, the surface grains of the Nb-Ti alloys could be best observed.

To shed light on the structure and chemistry of the specimens at the nanoscale, cross-sectional transmission electron microscopy (TEM) was applied. Characterisation was performed using a JEOL JEM-2200FS electron microscope (JEOL, Tokyo, Japan) operated at 200 kV. The TEM was fitted with an in-column Omega filter and a CMOS-based camera, TemCam-XF416 (TVIPS, Gauting, Germany). Images were captured utilising zero-loss filtering. Cross-sectional lamellae were prepared via focused ion beam (FIB) milling (CrossBeam 1540 XB, Zeiss, Germany). Before cutting, the samples were covered with an electron beam-stimulated Pt deposit, followed by an ion-stimulated Pt sacrificial layer to protect the surface. For qualitative elemental analysis, energy-dispersive X-ray spectroscopy (EDX) was performed in scanning (S)TEM mode utilising an X-Max^N^ 80 T detector from Oxford Instruments (UK). The data were processed with dedicated Aztec Version 4.0 software.

## 3. Results and Discussion

**Figure 2:**
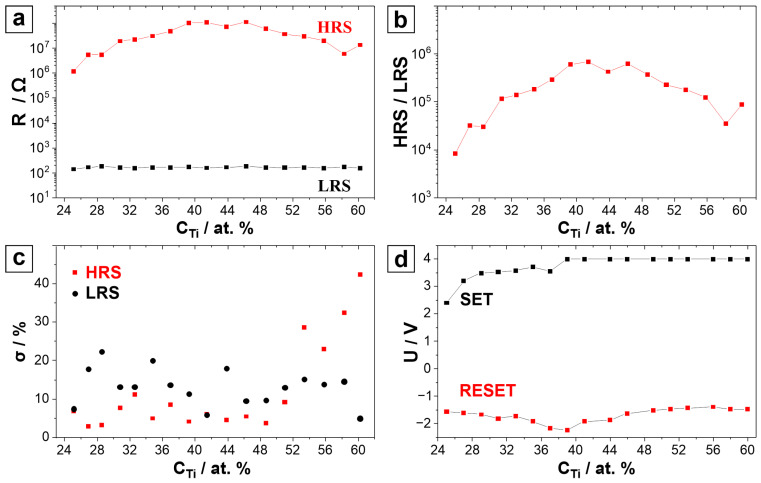
Compositional mappings in the Nb-Ti-based memristor library of (**a**) high and low resistance, (**b**) HRS/LRS ratio, (**c**) relative standard deviation (σ) values of HRS and LRS and (**d**) SET and RESET potential values measured at a 10 mA current compliance.

Using a combinatorial approach, it is possible to screen a large number of different materials efficiently. In this study, memristors anodically grown from different Nb-Ti alloys were analysed with a compositional resolution of 2.5 at.%. This led to the identification of 18 different main memristor clusters, which were distributed along the entire compositional range of the parent metals, spreading between 22 and 64 at.% Ti (see Figure 1). Vertically, along each cluster column, additional memristor clusters sharing the same composition were used for the statistical measurements. Figure 2a shows the high- and low-resistance-state values of the memristors along the Nb-Ti combinatorial library as deposited on the oxidised Si wafer. The evolution of the HRS can be correlated to compositional changes in the library, and a maximum region defining a plateau is observed for compositions ranging between Nb-39 at.% Ti and Nb-46 at.% Ti. Here, the measured resistance reaches high values of *R_HRS_* = 1 × 10^8^ Ω. The LRS remains rather constant along the entire Nb-Ti library, with low resistive values of *R_LRS_* = 2 × 10^2^ Ω. Since for most applications the ratio between the high and low states is relevant, it is presented in Figure 2b for all compositions studied along the library. As expected from the values of the individual resistive states described in part (a) of the figure, a trend similar to the HRS evolution is observed due to the constant LRS compositional behaviour. Thus, here, a plateau of maximum resistive ratio values in the compositional range of Nb-39 at.% Ti and Nb-46 at.% Ti is visible, where the ratios reach very high values close to 10^6^, which is beneficial for memristive performance.

To characterise the measurement precision, in Figure 2c, the relative standard deviations (σ) of the LRS and HRS measurements are presented as a function of the Nb-Ti parent alloy composition. The standard deviation values of the LRS are scattered between 5 and 20% throughout the library. Compared to these values, the HRS σ values indicate a much more accentuated scattering of experimental data after triggering the memristive SET process. Anodic memristors corresponding to compositions between Nb-24 at.% Ti and Nb-50 at.% Ti have reasonably low HRS σ values in the range of 3 to 12%, but memristors corresponding to compositions between Nb-50 at.% Ti and Nb-60 at.% Ti show much higher σ values in the range of 20–45%. This could indicate stronger instabilities in the mixed anodic oxides containing the highest amount of Ti under investigation, leading to switching variations among memristors from the same cluster. Additionally, device uniformity and device-to-device variations were calculated based on five representative retention measurements. It was found that device uniformity is more than 99% and device-to-device variation is less than 3% through the whole compositional spread, with no significant variations between each other.

In Figure 2d, the compositional dependence of the SET and RESET potential values is given. From the Nb-25 at.% Ti alloy to the Nb-39 at.% Ti alloy, there is an increasing trend for the SET values, which starts at *U* = 2.4 V and ends at *U* = 4 V, with a constant value of *U* = 4 V being retained until Nb-62 at.% Ti. In this compositional range, the constant potential value of *U* = 4 V is determined to correspond to the limiting current compliance of 10 mA which was reached at this voltage. The RESET value shows a decreasing trend from Nb-25 at.% Ti to Nb-39 at.% Ti. It starts at *U* = −1.5 V and reaches *U* = −2.3 V, where a minimum is found. From this composition forward, the RESET values start to increase, reaching a maximum at Nb-56 at.% Ti with a value of *U* = −1.5 V. It can be observed that both the SET and RESET values decrease at Nb-39 at.% Ti, the latter actually reaching its minimum. This would suggest that the anodic oxide corresponding to this parent metal composition has a low electrical resistance, which is in line with previous studies [38]. However, the electrical resistivity variation itself is likely not the only reason for the improvements in the memristive behaviour, and the anodic memristor based on Nb-46 at.% Ti was found to be the best performing along the entire compositional spread under study based on its HRS/LRS ratio and performance variability.

**Figure 3:**
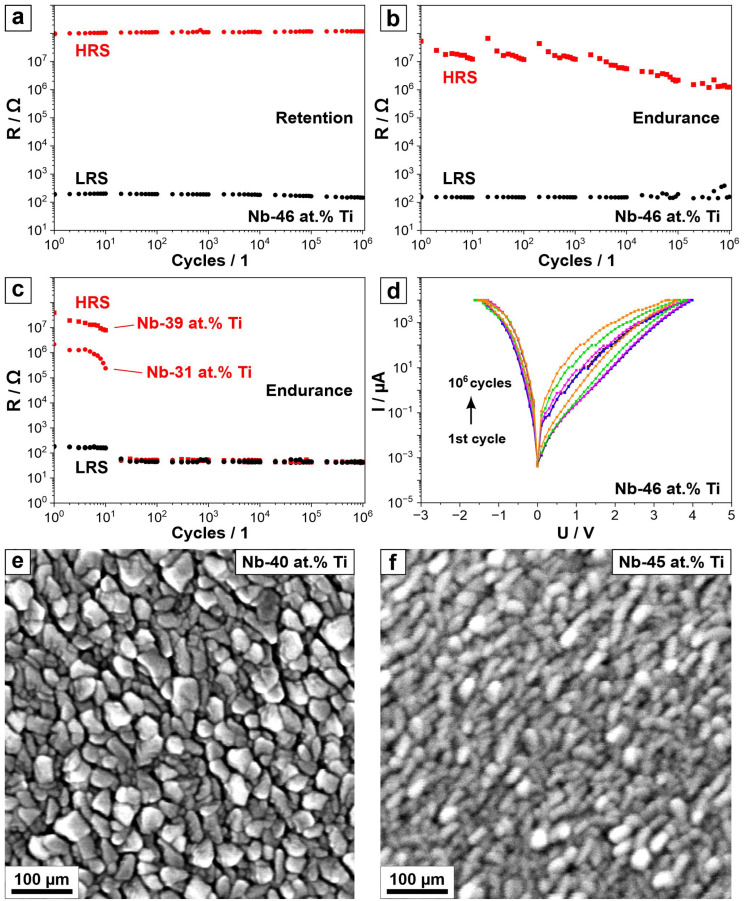
(**a**) Retention of the HRS and LRS values of the Nb-46 at.% Ti memristor. (**b**) Endurance of the Nb-46 at.% Ti memristor. (**c**) Endurance of the memristors for the compositions in a range of Nb-31 at.% Ti to Nb-39 at.% Ti. (**d**) *I–U* sweeps after each decade during the endurance measurement for the Nb-46 at.% Ti. SEM images of (**e**) Nb-40 at.% Ti and (**f**) Nb-45 at.% Ti metallic thin films.

Figure 3a,b show the selected retention and endurance data corresponding to the memristors based on a Nb-46 at.% Ti composition, while the endurance for the memristors in the Nb-31–39 at.% Ti compositional range is presented in part (c) of the figure. The retention of the entire compositional range was evaluated by measuring the device resistance after each switching procedure, up to 10^6^ cycles. The Nb-46 at.% Ti memristors also had a very good endurance of up to 10^6^ cycles. During the initial measurements, it was found that the memristors showed a volatile behaviour, as defined by them not retaining their resistive state when a (low) reading voltage was applied. Therefore, during the endurance experiments, the resistive states were read directly at the voltage values corresponding to the maximum and minimum resistance values. The HRS values showed certain instabilities after each *I–U* sweep performed at the beginning of each decade. The HRS values decreased slightly during each decade, showing higher values at the start and lower ones at the end of the decade. Additionally, these values decreased gradually during the whole endurance measurement. This behaviour is attributed to the thermal stress of the oxide leading to a decrease in its resistance, as previously reported [39]. While the endurance of the majority of the memristors from the library was still good after 10^6^ cycles, their endurance in the Nb-31–39 at.% Ti compositional range (see Figure 3c) was only 10 cycles. After 10 cycles, there was no more distinction between HRSs and LRSs but only a constant resistance value lower than the initial LRS. This phenomenon may be justified by an oxide breakdown, leading to a non-reversible switch to a LRS through a permanent current path in the active layer, producing a short circuit between the top and the bottom electrodes. Figure 3d shows the *I–U* sweeps measured after each logarithmic decade during the endurance test of the best-performing memristor. The evolution of the curves after each decade is consistent with the previous observation, where the HRS values change after each decade during the endurance measurements.

Examining the SEM images of the parent metal alloys obtained immediately after the Nb-Ti co-sputtering (see Figure 3e,f), a transition in the grain size and morphology is found at a compositional threshold between 40 and 45 at.% Ti, which confirms previous results reported in an earlier study [38]. This transition closely correlates with the plateau of the HRS seen in Figure 2a. Moreover, this also coincides with a reported stabilisation of the cubic structure for compositions above 45 at.% Ti [38]. Additionally, the transitional compositional range between 30 and 40 at.% Ti, where the cubic structure is unstable, correlates with the range where the memristors showed the worst endurance. This indicates that the previously reported surface structure and parent metals’ crystallographic compositional dependence may influence the electrical properties of anodic memristors. Considering all these parameters, Nb-46 at.% Ti is emphasised as the optimal composition for the best-performing memristor stemming from the library screening.

**Figure 4:**
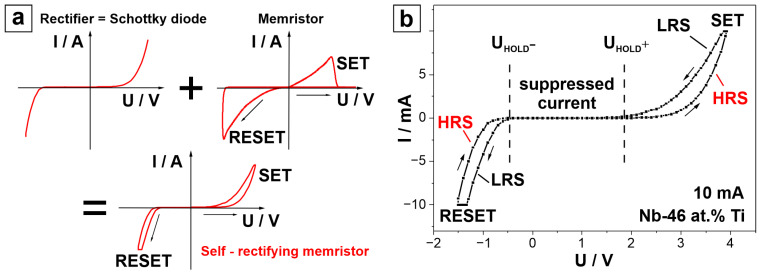
(**a**) Schematic representation of typical *I–U* sweeps for a rectifier, a memristor, and a self-rectifying memristor. (**b**) *I–U* sweep of the Nb-46 at.% Ti anodic memristor.

Figure 4a shows typical *I–U* sweeps from a rectifier, a memristor, and a self-rectifying memristor. The self-rectifying memristor behaves like a 1D1M device by combining a Schottky barrier formed at one of the MIM interfaces with the memristive active layer [6,25,27,31]. In this work, this is achieved intrinsically due to a fabrication procedure involving the direct anodic oxidation of the Nb-Ti alloy bottom electrode followed by Pt top electrode deposition. The most relevant role of the 1D1M device is related to its capability to suppress sneak current paths in crossbar array architectures. Combining this feature with the volatile nature of the memristors under study here, Nb-Ti anodic memristors may be very good candidates for neuromorphic computing purposes [24,25,28,40].

In Figure 4b, the *I–U* sweep corresponding to the Nb-46 at.% Ti anodic memristor is presented, and its similarity to the typical self-rectifying memristor behaviour is evident. At the start of the *I–U* sweep, the memristor is in a HRS. By applying a positive bias, the current starts to increase significantly only after approximately *U* = 2 V, which is defined as a hold voltage (*U*_HOLD_^+^). This is the voltage at which the current starts to increase following a suppressed state. After reaching an SET voltage of *U*_SET_ = 4 V, the memristor is switched to the LRS. Next, by decreasing the voltage, at *U*_HOLD_^+^ = 2 V, the LRS current is suppressed again. By further lowering the voltage towards a negative bias, the current starts to increase again at approximately *U*_HOLD_^−^ = −0.5 V until it reaches *U*_RESET_ = −1.5 V, where the LRS switches back to the HRS. The strong current suppression part of the *I*–*U* curve, defined by the two holding potentials (*U*_HOLD_), is a typical characteristic of a self-rectifying memristor. Self-rectifying behaviour is often attributed to the interfacial type of the memristors due to the formation of the Schottky barriers, which determine their switching mechanism [6,39,41].

In Figure 4b, the transition from the HRS to the LRS (and vice versa) is gradual. Previous studies confirm that this type of *I–U* curve characterises an analogue, interfacial type of memristive switching [6,20,42]. Interfacial memristors switch between resistive states due to the kinetics of the O species (vacancies and ions), which are distributed over the entire active interface. Together with the formation of Schottky-like potential barriers, the resistive states can be tuned by varying concentrations of O species [6,43]. Driven by the applied electric field, the accumulation of O vacancies near the interface results in a reduction in the barrier height, causing the system to enter a state of low resistance. Conversely, when the electric field depletes O vacancies from the interface, the barrier height increases, restoring the system to a state of high resistance [6,41]. The accumulation of O vacancies is likely induced by initial electron trapping at the Pt/oxide interface. This results in a negatively charged interfacial layer, leading to O vacancy accumulation in the depletion region to satisfy charge neutrality [39]. To investigate the source of the O vacancies, a detailed examination of the Nb-Ti anodic memristors was further performed.

**Figure 5:**
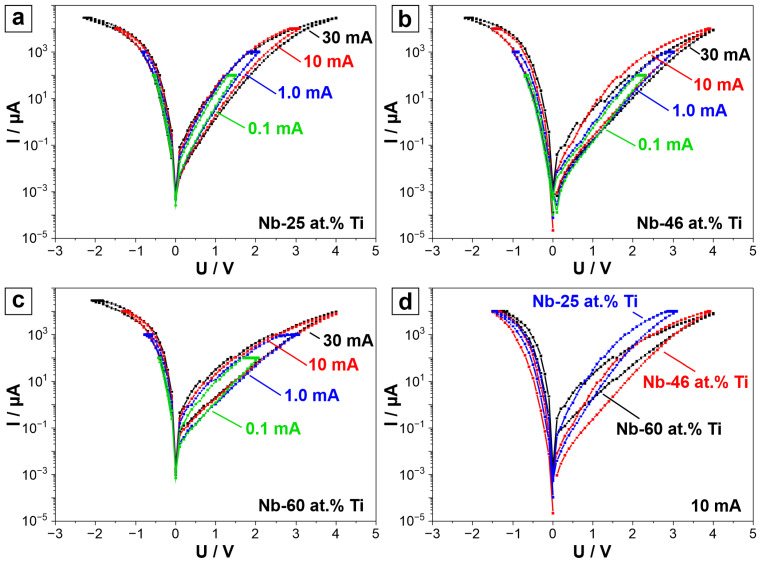
*I–U* sweeps (logarithmic representation) of (**a**) Nb-25 at.% Ti, (**b**) Nb-46 at.% Ti, and (**c**) Nb-60 at.% Ti memristors for current compliances of 0.1, 1, 10, and 30 mA. (**d**) Comparison of the Nb-25 at.% Ti, Nb-46 at.% Ti, and Nb-60 at.% memristors for a current compliance of 10 mA.

Following their discussed relationship to the parent metal alloy behaviour, the memristors along the library were classified into three compositional groups, with a threshold described by the best-performing device (Figure 4b). Figure 5a–c show *I–U* sweeps of representative memristors for each compositional group (with similar characteristics) at different current compliances, together with those of the threshold device. Comparing the *I–U* sweeps with different compliances for the same composition, it can be seen that the SET and RESET values change. Moreover, the LRS values change. This is usually characterised as multilevel switching, one of the most important parameters for neuromorphic applications. Although multilevel switching behaviour is often associated with a filamentary switching mechanism, where multiple concurrent conductive filaments (CFs) enable multiple resistance states [44], there have been reports of multilevel switching for interfacial, non-filamentary memristors [20,41]. The multilevel switching for interfacial memristors can be explained by a modulation of the interfacial O vacancy concentration [41]. Multilevel tunability provides the ability to considerably improve the plasticity of electronic synapse equivalents in artificial neural networks. Moreover, the multilevel switching tunability of the resistance levels provides the opportunity to store more than 2 bits in a single unit, which leads to designing higher-density and more robust neural networks [45,46]. Figure 5d shows the three representative memristors (based on Nb-25 at.% Ti, Nb-46 at.% Ti, and Nb-60 at.% Ti) measured at the same current compliance of 10 mA. It can be easily seen that the HRS, LRS, SET, and RESET values differ just as described before.

**Figure 6:**
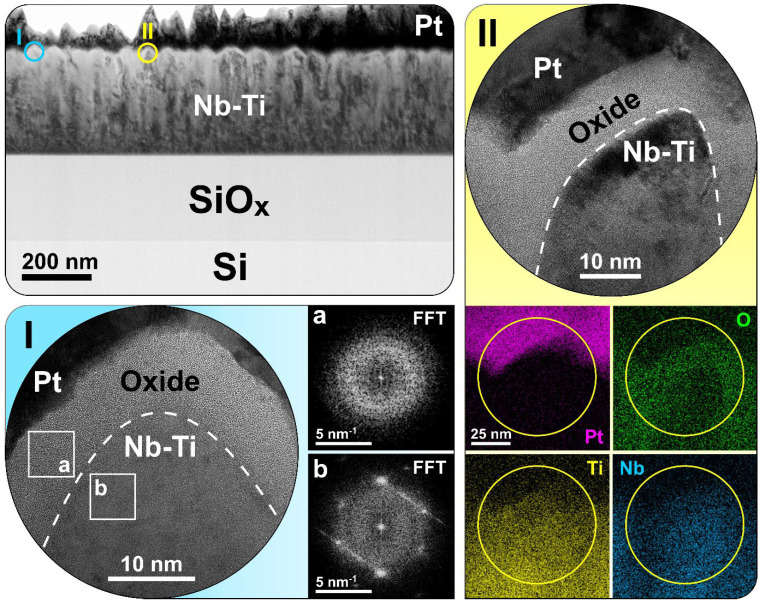
TEM investigation results showing a cross-sectional overview of the MIM structure on the substrate comprising the bottom (Nb-Ti) and the top (Pt) electrodes separated by an insulating anodic oxide layer. HRTEM imaging of regions I and II, together with FFT patterns (insets a and b) and STEM EDX mappings, is presented in the corresponding insets.

For deeper insights into the peculiarities of the MIM structure of the best-performing specimen based on the Nb-46 at.% Ti alloy after testing, additional TEM characterisation was fulfilled. A summary is provided in Figure 6. One can see that the Nb-Ti alloy film has a desirable thickness of approximately 300 nm (Section 2.1), a columnar structure, and a quite distinct wavy surface morphology with a roughness of ca. 25–30 nm. High-resolution HRTEM imaging reveals the homogeneous amorphous structure of the anodic mixed-oxide layer, while the parent Nb-Ti alloy remains crystalline (see FFTs of region I in Figure 6). It must be emphasised that no local variations in contrast, which can point to the formation of CFs, were observed in the specimens under investigation. This indirectly testifies in favour of the above-mentioned interfacial nature of the memristive effect for the Nb-Ti system. The mixed-oxide layer has an average thickness of only 10 nm and is twice as thin as the desired one according to the potentiodynamic anodization routine (Section 2.1). This phenomenon can be explained by the additional consumption of O upon oxide formation at the grain boundaries. The event can be readily seen in HRTEM and the corresponding elemental EDX mappings of region II (Figure 6). As a result, this “not fully developed” mixed-oxide layer seems to be sufficient for the good endurance and retention performance of the Nb-46 at.% Ti specimen (Figure 3a,b), but it could be the reason for the premature failure of other alloys, for instance, the Nb-39 at.% Ti and Nb-31 at.% Ti alloys (Figure 3c). According to the qualitative EDX, a noticeable component redistribution during anodization took place. While having an average of 46 at.% Ti in the parent alloy, one can observe the depletion of Ti in the crystalline grain “core” area, leading to a Nb/Ti ratio of 61/39. At the same time, due to the greater tendency of Ti to oxidise, its presence in the mixed-oxide regions at the surface and grain boundaries is significantly higher. The Nb/Ti ratio in these areas ranges from 41/59 to 49/51. For instance, one can clearly observe Nb depletion at the grain boundaries (see EDX mappings in Figure 6). Thus, compositional fluctuations lead to oxide stoichiometry variation, which may influence the O species kinetics.

This qualitative finding is in line with quantitative XPS analysis of anodized Nb-Ti alloys that previously revealed the oxide compositions change with the depth [47]. In particular, for the Nb-46 at.% Ti anodic oxide, it was found that the Ti amount increases from 46% in the parent metal to 52% in the mixed oxide. This indicates a gradient in the Ti (and complementarily Nb) content within the oxide, which is discussed from the point of view of different ionic transport numbers of Nb and Ti in their relation to O. Since Ti has a larger transport number than Nb, the mixed oxide has more Ti at the top, i.e., further away from the parent metal interface, as previously reported [38]. This has a direct effect on the stichometry of the oxides [48]. The gradient in the oxide elemental content results in suboxide formation, with direct implications for the number of O vacancies present.

**Figure 7:**
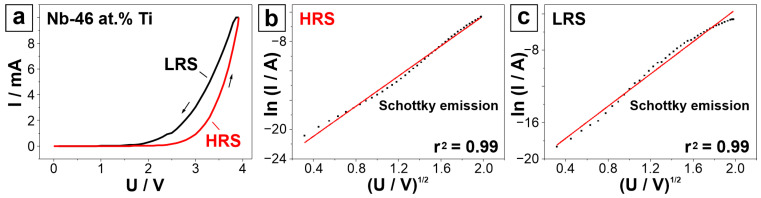
(**a**) Representation of the positive part of the *I–U* sweep with indicated HRS and LRS used for the current analysis. Current conduction analysis of (**b**) HRS and (**c**) LRS parts of the CV. ln(*I*/A) is plotted against (*U*/V)^1/2^. The linear plot represents Schottky emission as the predominant conduction mechanism. In subfigure (**a**), the arrows represent the direction of *I-U* sweep. In subfigures (**b**,**c**), the red line represents the linear regression plot, while the dots represent the experimental data from the *I-U* sweep.

To determine which conduction mechanism dominates the Nb-Ti anodic memristors, a current conduction analysis was carried out. In Figure 7a, the positive part of the *I–U* curve of the Nb-46 at.% Ti anodic memristor indicating the HRS and LRS parts is presented. These are further subjected to the current conduction analysis shown in parts b and c of the same figure. Plotting ln(*I*/A) against (*U*/V)^1/2^, a linear regression suggests Schottky emission as the predominant conduction mechanism [49,50,51]. This is observed for both the HRS and LRS parts of the *I–U* curve. Schottky emission is frequently observed as the predominant conduction mechanism in oxide materials and has already been reported for valve-metal-based memristors [50,52,53], including Ti/TiO_2_ [54]. This is not entirely surprising since Schottky, or thermionic, emission is the phenomenon by which thermally activated electrons are injected across an energy barrier into the conduction band of the oxide [50]. Therefore, Schottky emission is safely assumed to be the dominant conduction mechanism in the current study, which also supports the proposed memristive switching.

## 4. Conclusions

In this study, a combinatorial analysis was successfully performed on Nb-Ti-based memristors. Memristors anodically grown from different Nb-Ti alloys compositionally ranging between 22 and 64 at.% Ti were analysed. The highest values found for the HRS define a maximum plateau reaching 1 × 10^8^ Ω for compositions ranging between Nb-39 at.% Ti and Nb-46 at.% Ti. The LRS values remained constant during the entire compositional range, with values around 2 × 10^2^ Ω. Furthermore, the HRS/LRS ratio values follow the trend of the HRS due to the constant behaviour of the LRS, forming a plateau at HRS/LRS = 6 × 10^5^. The stability of the HRS and LRS during the writing process was studied, and it was found that there is a transition from less stable HRS values at Nb-50 at.% Ti to a more stable one for Ti concentrations below 50 at.%. Along the entire library, the anodic memristors showed good retention. The device endurance measured for the entire library was generally very good, demonstrating switching capabilities up to 10^6^ cycles, except for devices in the range of 31 to 39 at.% Ti, which reached their breakdown after only 10 cycles. This was linked to a transition in the compositional range where the cubic structure of the parent metal alloys stabilises, inducing a significant change in the surface structure morphology of the bottom electrodes, affecting the mixed Nb-Ti oxide growth and behaviour. Additionally, the plateau of the HRS (and HRS/LRS ratio) observed between 40 and 45 at.% Ti is interpreted as confirmation that the surface structure and the crystallography of the parent alloy influence the electrical properties of anodic memristors. All the analysed memristors demonstrated multilevel properties, which is a very important feature for neuromorphic computing. By analysing the *I–U* sweeps of the memristors, a typical behaviour corresponding to interfacial analogue memristors was found. Additionally, the Nb-Ti-based anodic memristors showed self-rectifying behaviour, which in applications is used for the sneak current suppression of crossbar arrays. The HRTEM analysis revealed the homogeneous amorphous structure of the active mixed-oxide layer, showing no CF formation. This allowed for the conclusion of an interfacial switching mechanism for the devices under study. The direct observation of the MIM structure cross section discloses the challenges that must be considered for the further optimisation of anodic Nb-Ti memristor fabrication. In particular, the parent metal alloy film has a substantial roughness which is transmitted to the active oxide layer, affecting the device’s stability and performance. The anodic oxide itself was thinner than intended; thus, the anodization parameters must be further adjusted while taking into account the additional consumption of O upon oxide formation at the grain boundaries and its penetration into the parent Nb-Ti alloy film.

Additionally, Schottky emission was determined to be the dominant conduction mechanism, confirming the proposed switching mechanism, which is based on the formation of the Schottky barrier. Finally, taking all of the measured parameters of the memristors with different compositions into consideration and their volatile and self-rectifying behaviour, the memristor based on a Nb-46 at.% Ti composition was chosen as a great candidate for usage in neuromorphic computing networks.

## Data Availability

Data will be made available on request.
